# Domestic cat management in the UK: learnings from a global perspective

**DOI:** 10.3389/fvets.2025.1610123

**Published:** 2025-07-22

**Authors:** Jenni L. McDonald, Dave J. Hodgson, Claire Roberts, Lauren Finka, Vicky Halls, Rae Foreman-Worsley

**Affiliations:** ^1^Feline Welfare Research Team, Cats Protection, National Cat Centre, Haywards Heath, United Kingdom; ^2^Centre for Ecology and Conservation, University of Exeter, Penryn, United Kingdom; ^3^International Cat Care, Tisbury, United Kingdom

**Keywords:** population management, community engagement, TNR, neutering, domestic cat, *Felis catus*, unowned cats, overpopulation

## Abstract

The overpopulation of domestic cats has the potential to result in negative outcomes for cats, people and the surrounding environment. A whole-population approach to management requires a system of services considering owned, shelter and free-living, unowned cats. Population management should also be considered at a localised level, with thought given to the unique populations of both cats and people in each environment. There is no simple, overarching solution to effective cat population management. Long-term management improvements require the addressing of root causes of overpopulation, rather than simply controlling the abundance of unowned cats. The role of rehoming organisations can be optimised by taking in only those cats that are suitable for rehoming and managing other unowned cats through community-level interventions. These approaches are beneficial for cat welfare, the welfare of cat carers and ultimately help more cats. Population processes, including reproductive output and survival of cats, and the carrying capacity offered by their environment, should also be critical considerations for the management of free-living, unowned cats. Compensatory effects, such as the movement of cats from neighbouring unowned or owned populations following population declines due to trap-neuter-return or rehoming, may contribute to the limited success of management programs. Education of cat carers around feeding and its effect on local carrying capacity is likely to be a valuable component of population management. Unrealistic expectations for the success of population management will be mitigated via better understanding of the population processes of domestic cats and of the attributes, attitudes and behaviours of people within local communities. It can take time for population numbers to reduce meaningfully via natural-cause mortality, and short-term population reductions can be misleading as populations may return due to compensatory processes. This wider understanding both within affected communities and for those actively participating in management is critical to developing practical solutions with realistic outcomes. Indeed, where there are owned cats or neighbouring free-living, unowned cats, then population management should be considered a permanent range of services that need to be sustained and adapted over time.

## 1 Introduction

Globally, free-living, unowned cats represent a complex socio-ecological problem. Although contexts for these cats vary greatly, in urban areas there are general welfare concerns regarding free-living, unowned cats. This includes not receiving appropriate care, leading to infectious diseases and elevated risk of outdoor-related injuries that remain undetected and untreated. Other concerns include a risk to public health through zoonotic disease (1, 2) or bites and scratches (3). Public perceptions of free-living, unowned cats can often be negative due to perceived nuisance behaviours such as noise, fouling and aggressive interactions with people (3) and their companion animals. This can present a severe welfare concern to these unowned cats if they are viewed as ‘pests’ and consequently subjected to inhumane management methods such as poisoning or injurious trapping. In rural areas, there may be additional concerns around their impact on livestock, including transmission of diseases. Free-living cats can also be a conservation concern, with impacts on biodiversity including predation, competition, fear effects, hybridisation and disease (4). While many of these concerns are applicable to both owned and unowned cats that are free roaming, they are elevated for unowned cats due to reduced or absent care provisions, such as preventative care or neutering, increased densities of unowned cats in localised areas and persistently living outside. Downstream effects of unowned cat overpopulation can impact rehoming organisations and cause welfare issues associated with overcrowding (5).

BOX 1 **Glossary of terms as used in this review**

*Subcategory*: A subdivision of the total cat population, including owned, unowned and shelter cats.

*Owned cat*: A cat that has a designated owner or owners, typically living in a household and dependent on humans for their needs. They are sometimes referred to as pet cats.

*Unowned cat*: A free-living cat that is not owned by any individual and may or may not be dependent on humans for food and shelter. These cats include socialised stray cats that previously lived with humans in domestic homes, unsocialised feral cats, and everything in-between.

*Shelter cat*: A cat that has been taken into an animal shelter, rescue or rehoming organisation. These cats may have previously been an owned or unowned cat.

*Overpopulation*: Where the numbers of unowned cats exceed the capacity of the local environment or community to humanely support them. This may manifest in negative consequences for the cat (e.g., poor health and welfare) and the wider environment (e.g., public health concerns, negative relationships with people, environmental impacts, impact on rehoming organisations).

**Figure 1:**
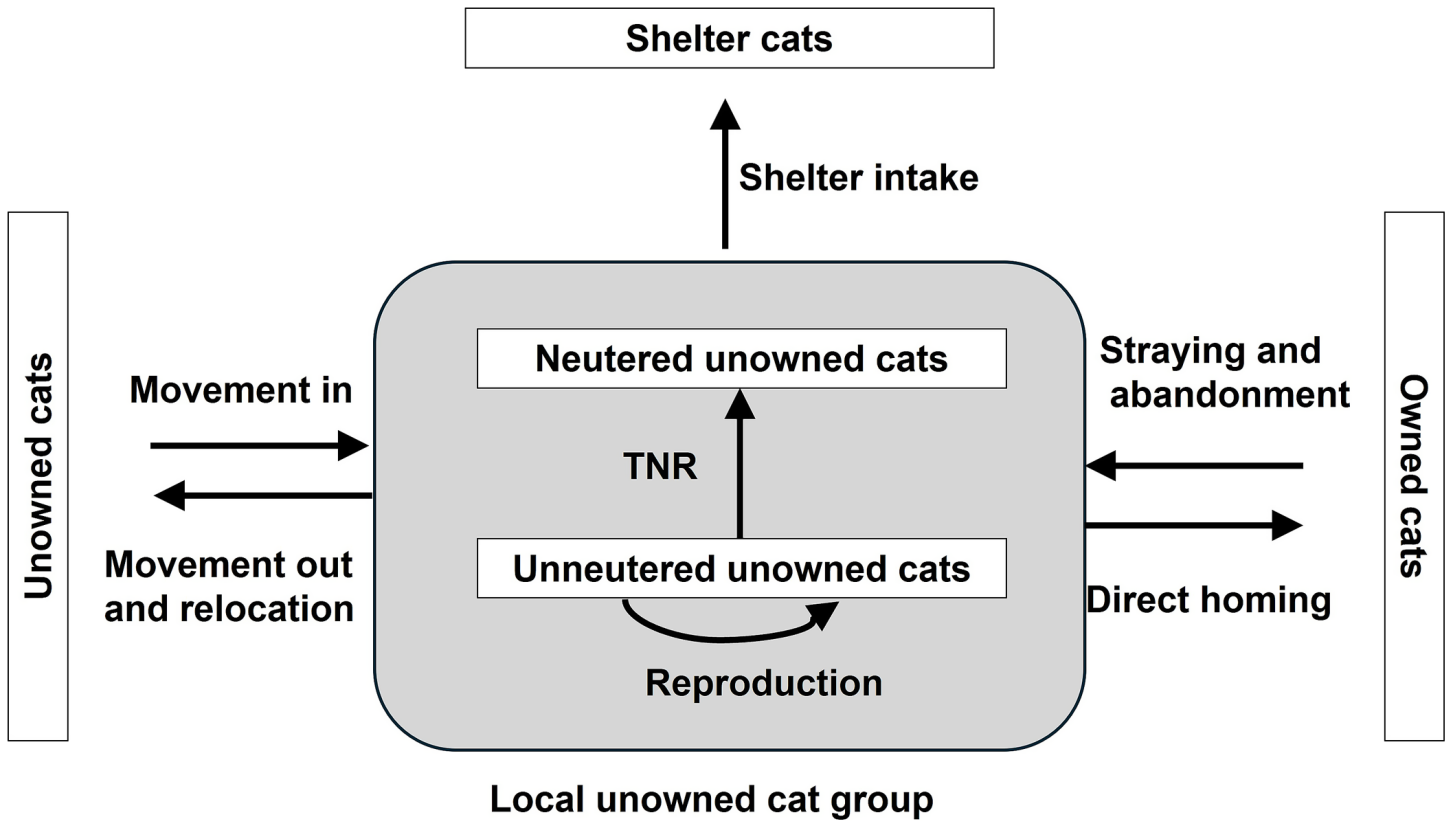
Interlinked nature of domestic cats living in different circumstances, highlighting how one local group of unowned cats is connected with owned, shelter and unowned cats in the surrounding area. Note, all groups will be interconnected but connections outside of the local unowned cat group are not shown for clarity purposes. Additionally, we do not distinguish between unsocialised and socialised unowned cats, with only the latter suitable for rehoming either directly or via a shelter.

Managing free-living, unowned cat populations can help mitigate against animal welfare problems, public health risks and conservation concerns. Other benefits include reductions in associated economic and emotional burdens that cat overpopulation can create. Typically, management programs aim to reduce the population size of unowned cats in local areas. Despite the frequency of cat population management programmes globally, there are few case studies that report on success and sustainability over time. A key consideration is the interconnected nature of subcategories of cats (e.g., owned cats, free-living, unowned cats, shelter cats as defined for the purposes of this review in Box 1), with cats’ circumstances subject to change and all unneutered cats having the potential to breed within the population. Cats commonly transition between distinct subcategories (Figure 1). Focusing on one intervention only (e.g., Trap-Neuter-Return (TNR), lethal control or shelter intake), rather than a combination of approaches, does not therefore account for the broader context of domestic cats, including the potential flow from the owned cat population due to unwanted litters, straying or abandonment, and movement of unowned cats into local communities. Increasing consensus discerns that programmes should address the sources of future unowned animals with a focus on root causes (6, 7), given that cat subcategories are interlinked and individuals can transition from owned to unowned, as demonstrated by previous modelling studies (8–11). Therefore, a combination of localised services to manage cats living under different circumstances is required to provide a whole population approach to population management.

Due to the interlinked nature of populations (Figure 1), desirable outcomes within communities may include (but are not limited to) those that promote responsible ownership (e.g., neutering and microchipping of owned cats; conducting pre-acquisition research; owned cat husbandry) and those that engage residents in accessing and driving services to manage unowned cats living on the streets (e.g., the reporting and/or neutering of unowned cats). Therefore, human behaviours towards cats are central to the effectiveness of population management (12) and highlight the need for context-specific management, shaped by the localised situation of cats, people and the environment.

In the UK, the problem is geographically heterogenous: significant numbers of unowned cats exist in some areas but not others. Current estimates of the density of urban free-living, unowned cats range from two to 57 cats per km^2^ (13). Demand for shelter space also varies geographically across the UK due to the presence of free-living unowned cats and the relinquishment of owned cats, but it typically exceeds shelter capacity in terms of both physical space and available cat carers, with many shelters consistently managing intake waiting lists (5). Given the significant geographic variability in the numbers of unowned cats across the UK, it is likely that the original sources of unowned cats will also vary across communities.

In the following sections we use global learnings to discuss humane cat population management in the UK context, rather than a review of cat population management which can be found elsewhere (14). We do not discuss non-surgical birth control, as there is currently no commercially available product suitable for unowned cats in the UK. Additionally, while lethal control is considered necessary in some environmental contexts globally, such as Australia (15), culling of unowned cats to support population management is not considered a routine option in a UK context. Indeed, multiple review papers have concluded that in terms of management the UK sits at one end of spectrum, supportive of TNR and not viewing predation as a threat, compared to Australian authorities occupying the other end of the spectrum (16, 17), with lethal control and containment laws in some areas. This is due to the lack of political and social acceptability of lethal approaches in the UK (17), along with different ecological and geographical contexts (17–19). This is in addition to general concerns around the impact on human wellbeing (20, 21) and cat welfare. Where there are identified conservation concerns to native British wildlife, such as a genetic and health risk to wildcats due to hybridisation (22, 23), competition (24) and disease transmission (24), non-lethal cat control remains the recommended action (24, 25). Consequently, our review focuses on the influential movements between owned, shelter and unowned cat populations, the role of community engagement, and the population processes (e.g., births, deaths, and immigration) that influence population dynamics. Our findings help to consolidate understanding, identify knowledge gaps regarding population management, and encourage practitioners to adopt an evidence-based approach grounded in realistic assessments of outcomes.

## 2 Culturally appropriate consultation, engagement and education

Successful and sustainable local implementation requires understanding of the behaviours towards cats within local communities and barriers to the adoption of desirable behaviours, such as neutering, to develop appropriate interventions such as training, modelling, enabling or education (12, 26, 27). However, the complexity of ensuring all views are accounted for means there is no one-size-fits-all solution, with inclusive engagement recognising that communities exist beyond geographical areas, and societal inequalities can drive disproportionate engagement. Barriers to engagement are diverse; attributes such as education, religion, resources, responsibilities (work and caring), language and disabilities may prevent people from taking part in community engagement processes. As seen in the provision of human health services, engagement approaches should be tailored to the needs of the local population rather than a national approach (28). Although the method of engagement is not the focus of this review, knowledge gained from information gathering such as focus groups, interviews or surveys can be used to improve community understanding around cats. Engagement may also involve stakeholders in devising solutions that are acceptable, utilising culturally appropriate information to empower individuals within communities.

Barriers to residents engaging with cat population management will often encompass broader community problems (26), such as limited access to health and human support services and financial difficulties. A one-welfare approach acknowledges these interlinks (29), but is not usually incorporated in cat management approaches (30). Community problems can also be exacerbated by broader economical and/or political circumstances in the UK. Given that the health and socioeconomic challenges that people face will directly impact on their ability to care for their cat, or those unowned in their community, a one welfare approach would be valuable (29), with the social determinants of human health linked to companion animal welfare (31). In these circumstances, community engagement could spark wider collaborations with other stakeholders and human agencies, such as housing authorities and foodbanks, with the aim to enhance welfare outcomes for both people and cats. Addressing broader community problems and offering social support has shown to be important for behaviour change in other contexts (32, 33) but is currently missing from traditional cat management approaches.

Every locality is a dynamic environment where humans, cats and other domestic and wild animals exist. No one plan fits all, therefore there is a need to consider culturally appropriate stakeholder consultation during all processes to achieve a sustainable management program (34).

### 2.1 Benefits of community engagement in terms of cat population management

Perceptions of cats are widely variable, which has been directly linked with the behaviours of UK residents towards them (26). Cats can be seen as valuable within their community, through their perceived control of vermin, or be seen as a negative influence (26). Differing views across community members can result in a reduced sense of community due to a lack of shared values. Engaging with communities while understanding and addressing the diverse perceptions of unowned cats can make management programs such as TNR more palatable and impactful. Not all residents will be supportive of having cats in their community. Reducing the impact of a perceived nuisance behaviour may be needed for cats to stay safely in the community and for staff and volunteers to operate safely and effectively. Many of these issues may be resolved by neutering interventions through reduction in undesirable cat behaviours as perceived by residents, such as vocalisations, urine spraying and fighting (35). However, this may be location specific, dependent on the densities and welfare of both owned and unowned cats, and the magnitude of resistance to them in the community.

Community engagement can address the causes of overpopulation by also focusing on the owned cat population. Assistive community-based cat programs in Australia that engage with residents to help identify and overcome barriers to neutering, opposed to traditional enforcement methods, have been found to improve compliance, and result in positive outcomes including reduced impoundments, euthanasia and complaints (30). Further studies in the UK and USA found that intensive community engagement efforts, combined with neutering programs, impacts the numbers of cats reported (26, 36) and numbers of cats neutered (37). Additionally, changes in resident perceptions can be positively affected including their perceived efficacy to find help for unowned cats (36, 37), encouragement of owned cat neutering and perception that animal welfare is improved (37). This enables a move away from a complaint-based program, where residents are reporting issues with cats, to one of positive volunteer involvement (38). Indeed, outcomes from a community engagement project within the UK included personal benefits for residents in the community and increased community ownership of the problem (36). In particular, community advocates are a potentially powerful group of people that know their area and are able to commit energy and time to assist. Although the long-term impact of such roles on cat welfare remains to be seen, volunteering in cat management programmes has been shown to increase participants’ capability and confidence to help cats, as well as provide personal benefits such as a sense of community, enjoyment and personal achievement (36). This may increase the chances of embedding sustainable, persistent, positive behavioural changes regarding cats (36). Stakeholder reengagement also builds local awareness and trust in staff and volunteers and can be a valuable source of information on hot spot areas to target, increasing the impact of management efforts such as TNR (26).

## 3 Owned cat management

Given the potential flow of owned cats into the unowned cat population, and vice versa, addressing overpopulation of unowned cats requires consideration of owned cats. Population management for owned cats includes both neutering to prevent accidental litters and prevention of abandonment and straying (Figure 1).

### 3.1 Encouraging timely neutering

Neutering domestic cats is generally seen as a positive welfare intervention for individuals and populations. Timely neutering to prevent accidental litters eliminates the risk of pregnancy and associated complications. Neutering has been linked to improved health outcomes [e.g., reduced risks of mammary tumour, carcinoma (39, 40)] and reduced undesirable behaviours [e.g., spraying, roaming, fighting (35)]. Consequently, neutered cats tend to have increased survival and longer lifespans than unneutered cats (41), along with the potential for physical and mental health benefits to owners due to a more positive human-cat relationship although this will be context dependent. In terms of population impacts, neutering owned cats not only prevents accidental unwanted litters in households, but in turn reduces numbers of free-living, unowned cats and demands for shelter space (Figure 1). Although the procedure itself has low rates of complications (42–44), the short-term impact of the stress and/or pain of the procedure on the cat can be a concern for owners. Additionally, neutered cats have increased risk of weight gain, obesity and diabetes (45–47), although this can be mitigated by proactive dietary management. Overall, most evidence highlights the benefits of neutering to individual cats as well as its role in providing a meaningful solution to the overpopulation of unowned cats.

An additional consideration is the age that cats are neutered. In the UK, routine neutering of owned cats from 4 months of age is recommended by veterinary and animal welfare organisations. Neutering cats after reaching breeding age can increase the risk of accidental early litters (48, 49), which risks an unnecessary burden on owners, impacting the welfare of both cats and people. Subsequently, this may affect the number of cats that are unowned, free-roaming or in shelters. Free-living, unowned cats tend to be more abundant in areas of economic deprivation in both the UK and New Zealand (13, 50). Internationally, lower neutering rates have been found for both the unowned (51) and the owned cat populations (52) in deprived areas. Modelling work based on the UK cat population finds that both neutering and age of neutering of owned cats are significant drivers of the numbers of unowned cats living in communities (9). Additionally, in Australia the targeted neutering of owned cats resulted in a decrease in shelter cat intake (30, 53). Therefore, encouraging prepubertal neutering of owned cats is a key management intervention for both owned and unowned cats.

#### 3.1.1 Neutering barriers

Internationally however, routine neutering of owned cats is not advocated for by all. In some countries, such as Germany, Norway and Sweden, routine neutering has been considered unethical and can only be done for medical reasons^1^ (54, 55). Whilst this is mostly applicable to dogs, it may arguably be relevant for some non-free-living cats, such as those kept indoors (56) where the risk of unwanted litters is reduced. In the UK, routine neutering is advocated and supported by veterinary and animal welfare organisations, and cat owners are also generally supportive (54). However, recent surveys estimate that 10% of the UK public may disagree with routine neutering and feel it should be done for medical reasons only (54). In some cultural contexts, neutering may not be supported at all. Indeed, although neutering prevalence of owned cats in the UK is generally high (85–87%^2^^,^^3^) with the average over the last 8 years estimated at 89% (see text footnote 3), the national average can mask geographic differences in prevalence. Therefore, understanding community level views will be key to effectively promoting neutering.

Barriers to neutering are likely to vary between and within stakeholder groups, e.g., owners, charitable organisations, or those who feed unowned cats. For owners, commonly identified barriers to getting their cat neutered, in both a UK and international context, include affordability, availability, accessibility, knowledge and perceptions (54, 57–60).

Veterinary care in the UK, including neutering procedures, has become more expensive. Although, reasons for increased veterinary costs are manifold and beyond the scope of this paper, they include the increase in the number of veterinary practices owned by corporate groups, issues of low recruitment and retention in the profession, global events such as Brexit and COVID-19 and increased inflation.^4^ Cost increases have coincided with a sharp increase in the cost of living for UK households.^5^ In 2024, only 62% of owned cats in the UK were reported to visit a veterinary practice routinely, with cost cited as a major barrier (see text footnote 2).

One key management intervention to overcome cost barriers is subsidised neutering. Subsidised neutering aims to preferentially target cats that are at risk of contributing to the unowned cat population, i.e., those who would otherwise not be neutered. However, while help with costs of neutering and other veterinary treatment is available via some animal welfare organisations in the UK, this is often restricted to specific catchment areas. It is currently unclear how this support is distributed across the UK.

Studies regarding the efficiency of neutering subsidisation vary in their results. A US study found that economic incentives for neutering resulted in a community-level increase in neutering prevalence, but did not observe any subsequent reduction in shelter intake or unowned cat populations (61). Communication of benefits may have indirect effects on neutering uptake within the wider community, particularly in areas that may not be eligible for the program but receive positive messaging. Additionally, messaging may result in positive social reinforcement resulting in promotion of regular neutering procedures (61), as has been seen within a TNR program in the United States whereby decreases in shelter dog intake were recorded, possibly due to the community engagement encouraging wider positive impacts on companion animal welfare (62). Thus, programs must combine a low-cost service with activity to convince people of the service’s benefits. Studies in the UK have found that cats attending a veterinary practice supporting a neutering campaign were more likely to be neutered before 6 months, compared to cats registered to the same practice before the campaign was established (63). Although this study cannot infer how or if this relates to a community level increase in neutering, it is promising that campaigns have the potential to shift the age of neutering.

Another key consideration in subsidised neutering is redemption of the neutering offer. A UK study found that just 65% of owners that applied for, and were offered, financial support in the form of a voucher towards neutering made use of it. Owners who received benefits were less likely to redeem vouchers than pensioners, students and people with low incomes (64). The reasons for this are unclear, but it highlights how more support than the voucher alone may be required for some owners. This lack of redemption may be linked to other barriers, such as physical accessibility for owners that have transportation challenges and/or limited resources such as cat carriers. Although in the UK veterinary coverage is generally high (relative to other countries), there will be disparities across communities regarding the time or distance required for owners to receive veterinary access, including proximity to veterinary practices and access to public transport. Therefore, the availability and accessibility of services need to be considered to account for the social and spatial dimension of healthcare access.

Other barriers to neutering include the availability of services, such as the capacity of veterinary practices and veterinary infrastructure to support neutering and carry out the procedure in a timely fashion. Waiting time for an appointment is a significant obstacle for owners in Brazil (58), and 28% of owners in the UK experience difficulty accessing veterinary care due to limited capacity at the veterinary practice. (See footnote 2) There may also be insufficient knowledge about where to seek veterinary care, meaning neutering procedures are delayed and the risk of accidental pregnancy increases. Additionally, in the UK, not all veterinary professionals carry out neutering at 4 months of age (65, 66), which is a particular concern when delayed neutering increases the risk of accidental pregnancy.

#### 3.1.2 Education about neutering

Misconceptions and low awareness around neutering and pregnancy in cats are commonplace. Misbeliefs include owners thinking a cat should have at least one litter and that related cats would not mate with each other (48). Many people are unaware cats can get pregnant at 4 months of age (48). This lack of understanding around the reproductive behaviour of cats can result in unplanned litters even if owners intend to have their cat neutered. Some owners are not supportive of neutering generally, driven by cultural or social factors, meaning the impact of education programmes may be limited if not addressing variations in cultural perspectives or potential language barriers.

Given cats are prolific breeders, tackling education around neutering for owners will always be a key intervention where owned cats are free-living. At a local level, community awareness campaigns and interventions must approach the issue of neutering in a way that is consistent with cultural, social and economic circumstances. Increasing levels of owner understanding around cat pregnancy and neutering benefits can be accomplished in different ways, and occur at every stage of ownership, including pre-acquisition, acquisition, kittenhood and adulthood. Veterinary surgeons are generally seen as a trusted source of information and play a key role in promoting neutering as a responsible part of cat ownership, as well as dispelling misconceptions and myths. However, interventions should also consider other routes to increasing owner knowledge of neutering through avenues such as community advocates, welfare organisations, corporations, mass marketing or social media, with the exact route tailored to the target population.

### 3.2 Reduce abandonment and straying

Preventing owner abandonment of cats and kittens can reduce the numbers of owned cats flowing into established or new unowned populations. Education and support for owners may reduce the likelihood of abandonment, relinquishment or straying. Potential owners should be equipped with appropriate information prior to acquisition to determine the best route to acquiring a cat, such as using the kitten checklist,^7^ and have realistic expectations around cat care and costs to ensure they are able to provide for their cat. Education around appropriate socialisation of kittens for shelters, breeders and owners should increase the likelihood that cats are adjusted to living in a household later in life (67, 68). Additionally, although there is limited evidence in this area, effective matching of cats to households is likely to be beneficial, ensuring both cat attributes and owner circumstances are considered during the rehoming process.

In the UK the majority (70%) of owned cats have some form of outdoor access; this is higher than that seen in other countries such as the United States, Canada, Australia and New Zealand (69). There are also no containment laws within the UK, which differs to some international contexts such as areas within Australia (6). Owner decision-making behind whether a cat has outdoor access is linked to multiple factors (69, 70) with motivations for keeping cats indoors differing between countries (69). Generally, in Europe (including the UK), the main reason for keeping cats indoors is related to perceived cat welfare benefits, with very few owners (1.4%) considering the impact of cats on wildlife (69). Whereas the impact on wildlife and preventing cats from hunting are a greater priority for cat owners in the United States, Canada, Australia and New Zealand. While a full review of the pros and cons of providing outdoor access is beyond the scope of this paper and is provided elsewhere (69, 71), we recognise that different lifestyles have the potential to contribute to overpopulation. Outdoor access may increase the risk of unintentional breeding and straying, with stray cats entering UK rehoming organisations (5). However, indoor-only cats may exhibit more “undesirable” behaviours from the owner’s perspective and may be at increased risk of abandonment or relinquishment (72), with behaviour problems cited as a key reason for relinquishment in the UK (73). Therefore, in the UK context, a holistic approach to support cat owners with cats of different lifestyles is required to reduce the likelihood of owned cats becoming unowned. This includes owners having easy access to behavioural and veterinary support to recognise and provide for the individual needs of cats, prevent escalation of perceived cat problem behaviours and interventions at times of crisis.

Effective microchipping programmes can reduce the number of unowned cats due to straying by enabling the reunification of cats and owners. At the time of writing, microchipping of cats is a legal requirement in England, with calls for similar laws in Scotland, Wales, and Northern Ireland. Compulsory microchipping of cats in England came into force in June 2024 with a significant rise in the proportion of cats microchipped in the run up to the legislation (see text footnote 2). Compulsory dog microchipping was introduced in 2016. Although studies are limited, a study of one local authority found the policy significantly improved the rates of return of stray dogs to their owners by the council (74). However, legislation alone is not the only consideration: the number of dogs microchipped has subsequently declined in 2024 (see text footnote 3). The reasons for this are unclear. It is possible that declines in microchipping are due to a lack of awareness of the legislation among new pet owners. External circumstances such as the rising cost of living (see text footnote 5) and the increased cost of veterinary treatment^8^ impacting owners and their ability to pay for veterinary care may also play a role. Additional considerations include ensuring contact details associated with the microchip are up to date, and that information is accessible through easy access to scanning equipment and central or interoperable databases.

## 4 Unowned cat management

TNR in its many forms is the most common management strategy proposed by animal welfare organisations and forms the basis of unowned cat management in the UK. There are many variants of TNR practiced by cat advocates. Some programmes include the removal of unowned cats from the community for adoption, such as kittens and socialised cats, whereas others are focused specifically on neutering and returning cats to their original location.

TNR is the subject of much debate between different stakeholders, and this is reflected in the variability in the published research on the topic (30, 75–78). In some countries, including New Zealand and most states of Australia, feral cats are controlled within the context of pest management and TNR is illegal or unsupported, with culling as the predominant recommended strategy for feral cats that live in the wild without human contact. Globally, some conservationists and animal rights groups consider TNR practice unacceptable for very different reasons, using terms such as “biological littering” (79) or “Trap Neuter Reabandon” (80) respectively. Anti-TNR sentiments are not as prevalent in the UK, however it is important to be mindful of the concerns of different stakeholders.

The evidence around population-level benefits of TNR is mixed, and there is much debate as to its effectiveness, with both supporting and opposing evidence (62, 76, 81, 82). It may also be reasonable to assume (as is the case in other fields of science) that fewer unsuccessful programs are published, opening up the potential that not all TNR programs have a population-level beneficial impact. Polarised thinking on TNR is potentially unhelpful and it is inaccurate to assume the effectiveness of TNR and the outcomes for cat welfare are uniform. Success depends on a range of diverse factors and their interplay, including implementation, context and expectations of success.

To understand why some TNR programmes are unable to bring about long-term reductions in numbers of unowned cats, we discuss the population processes that can curb the positive effect of TNR, and highlight situations where TNR is not an appropriate intervention.

### 4.1 Population processes

Free-living, unowned cats rely on environmental resources just like other wild animal populations. Therefore, where food is provided directly or indirectly, it has the potential to increase the number of cats that can be supported, termed the carrying capacity of the environment (Box 2).

BOX 2 **Carrying capacity**

Carrying capacity is the number of animals an area can support. The number of free-living, unowned cats in an environment will depend on the number of resources available. This is why unowned cats are largely solitary with large territories in non-urban areas, compared to the high densities of unowned cats seen around feeding stations where resources are provided, or in urban areas where there are more human-food scavenging opportunities. In a socially connected environment, increased resources result in increased densities.

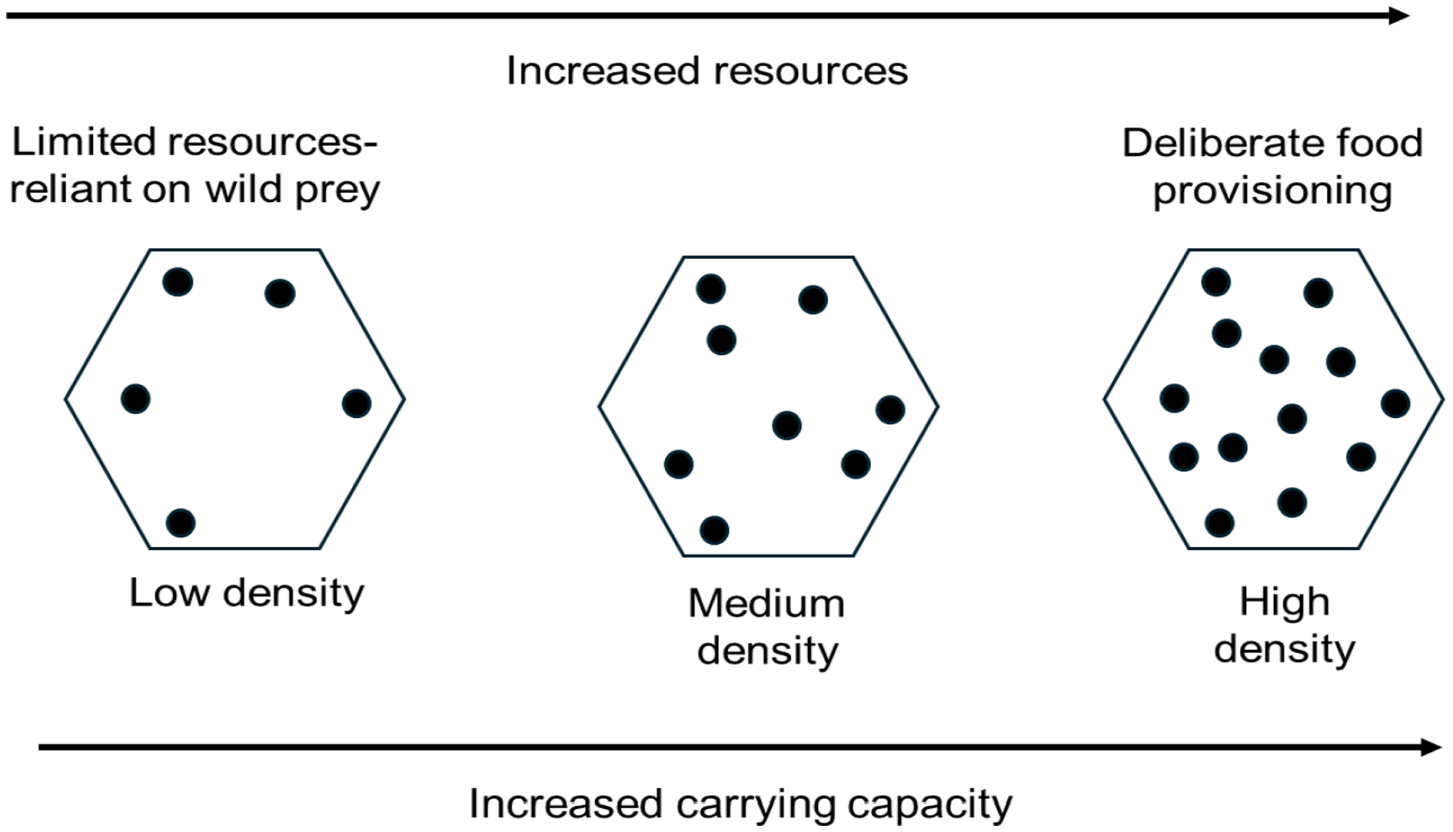

When populations decline due to removal or reduced reproductive output as a result of TNR, but levels of food sources remain constant, decreased cat density leads to increased resource availability. This can increase fecundity (for non-neutered cats) and survival, as well as promote movement into an area. These mechanisms can either partially or fully negate the impact of any management effort.

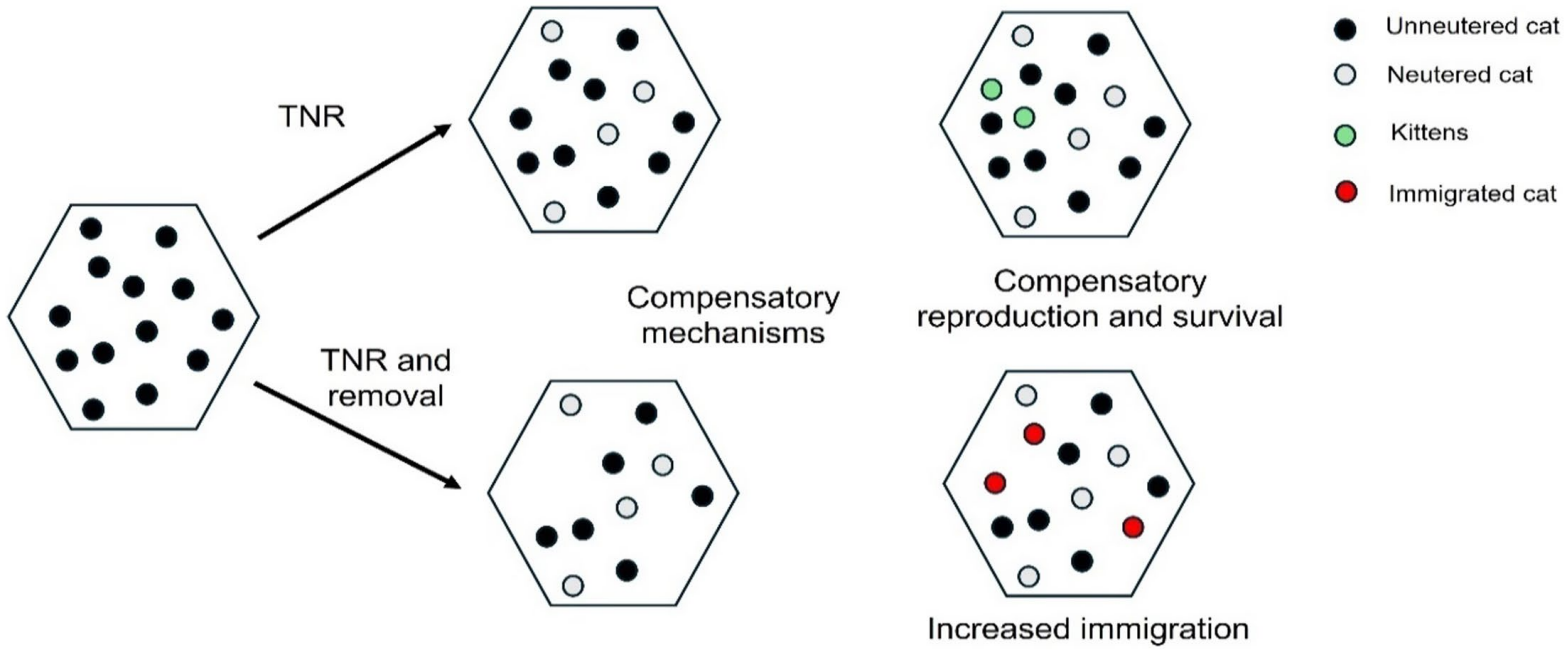

Generally, the low success of TNR programs is associated with the immigration of cats from neighbouring unowned cat populations, or increased straying or abandonment of owned cats (83–85), meaning TNR is seldom a one-off intervention. Additionally, there is some evidence to suggest that neutered cats, with reduced territorial aggressiveness, are more likely to accept new arrivals (35), again reducing any positive local impact of TNR that may have come about by natural attrition. A ten-year study of TNR in Rome was found to reduce the population size in colonies but concluded that the program was “a waste of time, energy and money” if abandonment of owned cats could not be stopped (7). Broad-scale effort, involving the wider community and over multiple years, is required to have measurable impact (38, 86). Indeed, TNR campaigns that have found a positive impact of stabilising or reducing population densities are those which are targeted, long-term and of high intensity (87) and/or linked with other interventions to prevent abandonment (88). The potential for colony extinction has also been demonstrated in a case study in the United States following 17 years of TNR (89).

Although estimates vary, general ecological studies suggest a significant proportion of female animals [90% (90)] would need to be neutered to overcome the compensatory effect of rates of immigration of non-neutered individuals. Cats may present even more of a problem due to their highly fecund nature (90). Even with high rates of neutering over contiguous areas, positive impacts can be limited. For example, a study in Israel found populations increased when TNR was carried out in distinct localised areas, presumably due to a continued influx of cats, despite 80% of cats being neutered. The same study found that city-wide neutering of over 70% of cats was needed to bring about population decline. However, this was also limited by a compensatory increase in reproduction and survival, likely due to increasing food availability, decreasing resource competition and reduced agonistic behaviour (84). A modelling study that evaluated two county TNR programs in the United States concluded that the populations did not decline and that higher rates of neutering (between 71-94%) were needed (91).

Some successful studies have included the removal of free-living, unowned cats from the environment, through rehoming or euthanasia of sick cats. Removal of individuals has the added benefit of being able to reach a target population size more quickly, instead of relying on natural attrition which is a much slower process. Rehoming paired with TNR has been shown to reduce shelter cat intake (62) and be effective at reducing populations (38, 88, 92), however short-term benefit may not be maintained if there is no continued effort to reduce the breeding of cats in the wider area. There are clear parallels in culling studies that reveal rapid replacement rates in some areas when cats are removed (93, 94). Culling can inadvertently lead to population increases (93, 94) and instability in population structures (95), both of which has been found to have negative outcomes, such as increased disease, in other wildlife populations (96, 97). Thus, removal effects can be counterintuitive without a range of other services in place. Additionally, careful consideration needs to be given regarding the suitability of free-living, unowned cats to be homed to domestic environments as this has the potential to have a downstream effect on cats in shelters. Many free-living, unowned cats have had no, or very limited, social experiences with humans, and thus potentially a limited capacity to adapt well to future environments that require close cohabitation (98). This lack of previous socialisation to humans is likely to compromise the wellbeing of these cats when housed within a shelter environment (98, 99) and attempts to human-socialise unfriendly and fearful cats beyond their ‘sensitive period’ (i.e., 2–7 weeks of age) has the potential to induce the experience of ‘learned helplessness’ [see (98) for a more detailed review]. Taking these cats into shelters not only compromises their welfare and increases their risk of stress-linked sickness behaviours, but also transfers the overpopulation issue into shelters, with these cats taking longer to be homed (100, 101). When shelters are at capacity, overcrowding can lead to disease outbreaks, and a lack of resources make it difficult to maintain minimum standards of care. This not only compromises the health and mental well-being of cats but also that of the people that work with them (102).

With these limiting factors in mind, successful programs highlight important predictors of success, which include intensity of operations (long-term and spatially contiguous), community engagement, consideration of resources and pairing TNR with cat removal, through rehoming of suitable cats and euthanasia of sick cats.

### 4.2 Contextual limitations and alternative approaches to TNR

There are some circumstances where TNR is not a viable solution. In terms of feasibility, there is a need to consider legality. Unlike elsewhere, such as some Australian jurisdictions (103), TNR is legal in the UK. However, land ownership may be a barrier with permission from private landowners a prerequisite to any population management activities. Additionally, TNR may not be feasible due to concerns regarding the health and safety of volunteers, such as unsafe terrain, lone working or restricted areas. Also, veterinary capacity and adequate processes should be in place to support neutering at a fast pace and for the long-term, e.g., flexibility with drop-offs, prepubertal neutering and tailored decision-making to best support the needs of individual cats.

There are also areas where TNR may not be appropriate for either the cat or other animals, including humans, in the surrounding environment. Some areas may be dangerous for the cat to return to such as hazardous environments or due to high levels of negativity towards cats that cannot be overcome via community engagement and nuisance management. TNR may also be inappropriate where neutering would not resolve a localised problem, such as in protected areas where predation poses a risk to wildlife. Although this is not as prevalent in a UK context compared to internationally, there may be localised areas where free-living cats are not appropriate as they represent a disproportionate risk to wildlife, such as in areas that ground nesting birds inhabit. Additionally, it would not be desirable to return cats to the same environment where there are concerns relating to public health such as a high prevalence of zoonotic pathogens and/or concerns around environmental accumulation of faeces and water pollution (104). In instances where the environment poses a risk to the cat and/or the cats pose a risk to the environment, trap-neuter-relocate may be more appropriate, providing cats are relocated to safe and suitable environments.

## 5 Shelter cat management

Cats residing in shelters are estimated to be the smallest subgroup of the UK cat population (9). Although accurate estimates are lacking, current approximations suggest UK shelters host an estimated 0.2% of the total population of cats at any one time (9). Targeting resources and intake is necessary to ensure cats are only taken in that can be cared for well. This subsequently improves population impact and safeguards the physical and mental health and wellbeing of cats and those that work with them. It may be assumed that shelters are a solution to the cat overpopulation problem, however their limited capacity to house cats and their inability to address the root cause of the problem (Figure 1) mean their role is limited in terms of overall population management. While a full appraisal of optimal shelter management is beyond the scope of this review, to improve the positive population impact shelters can have, there is need for education and training to ensure they only take in cats suitable for a home environment, to promote a culture of adoption and maintain throughput. The concepts discussed below are derived from the concept of capacity for care from the US (100) and the cat friendly homing principles of International Cat Care^9^ and aim to ensure shelters are working at optimum efficiency.

Ensuring a culture of adoption, whereby only cats that are formerly owned, or considered suitable to be owned in a home environment, should enter care (including homing centres and foster sites). Many free-living, unowned cats are not suited to cope well in confined environments or in close proximity to humans and rehoming them would compromise their welfare, exacerbate shelter overcrowding, and slow down the rehoming rate. Such cats should be managed through community-level interventions such as TNR or found alternative free-living environments to be relocated to that suit their needs (see text footnote 9). We note that other shelter management practices may also be important considerations. Such as return-to-field approaches (6, 105) where cats within a shelter that may be at risk of euthanasia are returned to their original locations. In the UK, most cat shelters are operated by charities and NGOs, which differ to government run shelters or animal control that are present in other countries. Consequently, TNR is routinely practiced for unsocialised unowned cats, capacity-related euthanasia is not overtly carried out and shelter euthanasia rates are typically lower than that observed globally [3–13% in the UK (5, 106, 107)]. Therefore the applicability of return-to-field of cats that have come into a shelter environment may be limited but is of relevance on occasions where cats are found to be unsocialised after entering a shelter and when returning them to their original location is feasible.

Additionally, shelters should operate at an optimum, not maximum, occupancy, accounting for physical space and capacity for care provision. Although it may seem counterintuitive to reduce occupancy, evidence suggests that this can increase adoption probability (100), which directly relates to a decreased length of stay (101), a reduced mortality rate (100), and fewer cats requiring infectious disease isolation (100). In contrast, where open intake policies are in place, overcrowding can result in a substandard quality of life for those cats due to increased stress, disease and consequently increased rates of euthanasia. Problems of overcrowding can increase compassion fatigue and emotional toll among shelter staff (102). As cited in an Australian study, the effect of overcrowded shelters and euthanasia of cats on staff included increased susceptibility to post-traumatic stress, depression, substance abuse, hypertension, sleeplessness and even suicide (92). Additionally, open intake requires the support of charitable donations from the public, potentially limiting operational activities in other areas or the wider community, resulting in fewer cats helped overall. Internationally, some areas have seen a move from immediately taking a cat into a shelter to a strategy of connecting residents with resources to keep cats *in situ*, which is found to be beneficial (62).

It is expected that adopted cats will already be neutered or will be neutered by adopters when reaching the required age or weight. Pregnant cats can also be neutered humanely and safely, preventing the expensive need to care for neonates. Kittens born in care contribute to an increase in the numbers of cats requiring homes, adding to problems of overcrowding, and may be inadequately socialised to successfully thrive in a home environment.

## 6 The application of evidence

Resource allocation for population management must be carefully considered to ensure maximum impact to benefit cat welfare in the long-term, particularly as charitable organisations are restricted financially. This may sometimes not directly align with reactive management strategies that strive to improve immediate welfare concerns (11). The primary questions for those investing in population management programs include when, where and how much effort, time and money should be expended and distributed. Learnings and considerations from the wider literature on domestic cat population management, aside from the critical importance of community engagement covered previously, are as follows:

*Targeting female cats is likely most effective*. As a single male can impregnate numerous females, castration is unlikely to have much effect on population-wide reproductive rates, if any, as castrated males will be out-competed for access to females by intact males. This is likely more relevant for free-living cats in geographically connected landscapes, where natural immigration of intact males will occur, rather than socially isolated environments such as oceanic islands, although models of such closed populations also highlight the importance of female-focussed neutering efforts (108). Furthermore, beyond population management, neutering may confer additional individual-level benefits, such as improved health outcomes (40, 41) and behavioural benefits (35, 109). Therefore, there is balance between maximising long-term cat welfare via effective management and the short-term benefits to individuals.
*Target younger cats to prevent accidental litters.* Modelling studies have demonstrated that younger neutering of owned cats is more effective at reducing unowned cat population numbers (9). However, there is no empirical evidence on the effect of reducing the age of neutering in unowned cats. In the absence of UK literature, global studies of unowned cat demographics find a high probability of mortality in early life (110–113). Consequently, there is a risk that early-life neutering may be wasteful of veterinary and charity resources. Additionally, body weight is a key determinant of feline sexual maturity (114), thus if unmanaged unowned cats are in poorer condition than owned cats (115), the benefits of neutering younger cats might be negated. This will be context-specific with more research needed in this area.
*Consider timing of interventions.* Cats exhibit distinct seasonality in births (116), thus ecological theory suggests that conducting population management at the wrong time may be inefficient. This may be less relevant for owned cats who live indoors some or all of the time, thus may be less impacted by environmental mating triggers such as temperature and daylight. Although there is currently no evidence of the relative benefit of timing of interventions versus timing of age of neutering, for owned cats the focus on early neutering may be more impactful. However, for unowned cats, the optimum time is likely to be when a localised population is at its smallest in advance of the breeding season (117). This will enable the highest prevalence of neutering for the least amount of resource, and increase the chances that cats likely to contribute to the breeding season are neutered. For example, a target of neutering 80% of all cats is more easily reached in a smaller population, instead of immediately following kitten season when the population is at its largest.
*Consider spatial targeting of areas.* It is best to target areas where the need for management programmes is the greatest, either due to persistent overpopulation of unowned cats, in areas where cats may be a conservation concern or where cats are not tolerated by the community. However, targeting based on numbers is challenging due to the lack of availability of local data for shelter and free-roaming animal populations. Whilst current data highlights there is an increased likelihood of unowned cats in economically deprived areas (13), spatial coverage is incomplete. Data gaps are further complicated by difficulties determining whether a cat is owned or unowned. While misidentification in population estimates can be overcome through bespoke modelling approaches (118), the need for local data can require significant resources in the form of time, person power, and analytic capabilities. In the absence of an understanding of unowned cats and their distribution across the UK, or means to accurately measure their numbers, it may be that proxy measures are used to guide targeting such as human-sociodemographic characteristics, deprivation indices (13, 50), areas with high-levels of cat-related calls or high demand on shelters to take in unowned cats.
*TNR needs to be high intensity, targeted to the broadest area feasible and a long-term endeavour.* Neutering few cats, *ad hoc*, from an area with high numbers of free-living, unowned cats is unlikely to have any meaningful population impact, as compensatory processes (increased reproduction, survival and movement of cats into the area) will outpace neutering efforts. Therefore, TNR projects need to aim to neuter high proportions of cats (70-94% [10, 11, 84, 90, 91)] over a large area, at high intensity, to counteract compensatory effects. Modelling studies have highlighted that high intensity TNR from the outset produced lower population abundances and fewer preventable deaths then a lower intensity scenario that spreads costs over time (10, 11). Such high intensity work necessitates financial resources, veterinary capacity and effective trapping protocols, making the maintenance of such a project challenging and costly, as previous projects have found (84).
*Ensure that supplementary feeding of cats is partnered by neutering advice and practice.* In the UK, feeding unowned cats is a more common practice than neutering unowned populations (36), with many people feeding cats they do not own (see text footnote 2). Although well-intentioned, feeding cats means the environment can support more unowned cats in terms of resources (Box 2), resulting in negative welfare outcomes such as intercat aggression or the spread of infectious diseases or parasites as cat density increases. Therefore, there is a need for community awareness campaigns and engagement to encourage neutering where feeding is taking place. *Ad hoc* feeding should also be discouraged where cats are in visibly good condition, and it is uncertain whether cats are unowned or originate from a domestic home. Feeding stations for large colonies of unowned cats are not as common in the UK as they are internationally. These feeding stations are generally not recommended as they may attract greater numbers of cats into the area (119, 120), in addition to other wild species (121), increasing the risk of conflict and disease which is counterproductive to TNR efforts.
*Reduce food sources in line with population reductions.* Where cat management is taking place it should be paired with a reduction in food sources to reduce carrying capacity in line with natural attrition or removal activities, maintaining any positive impact (122). To achieve this, cat carers should be incorporated into unowned cat population programmes to maintain neutering rates and control feeding, to allow adequate, but not excessive, food provisioning for cats in the target population. For example, people that provide food for cats could feed them at specific times, ensuring resident cats get adequate provisions and remove any excess so food is not left out in the environment. More research is needed to understand how to bring about positive human behaviour change in this area.
*For overabundant populations, a combination of removal (if appropriate for the cat) and TNR would shorten the time necessary to reach a target population size.* Removal of free-living, unowned cats paired with TNR is generally seen as more effective in the short-term at reducing unowned cat population numbers compared to TNR alone (10, 88). This is because with TNR-based approaches cats are not removed from the population until they die at a later time point, which contrasts to the direct removal of cats where the population declines immediately. However, this reduction in numbers may not be sustainable if interventions do not target compensatory effects. Additionally, considerations should be given to negative downstream effects on cats in shelters due to both overcrowding and slowed throughput if taking in cats that are not suitable for homing. Therefore, it is worth taking time to make informed decisions on shelter intake (see text footnote 9), as subjective assessments, although well-meaning, can be detrimental to both cat welfare and population management if affecting shelter capacity.

## 7 What does success look like?

The management of cat populations and desired outcomes lacks a standard definition of success, with different studies using different measures (Table 1). While an evaluation framework of success is beyond the scope of this paper, it has been found to be needed in other management areas such as conservation translocations (123). Different contexts of communities, the pertinent issues and stakeholder interests are likely to guide expectations. Project success may be defined and evaluated broadly as biological and ecological, social and/or methodological. Biological and ecological elements include the demographics, behaviours, health and welfare of cats and other animals. Social criteria include the health, behaviours, perceptions and attitudes of local householders. A methodological lens evaluates the process itself, which may be in terms of feasibility and engagement with the programme (see Table 1 for examples).

**Table 1:** Examples of different measures of success in cat population management studies.

Overarching lens	Target population	Measure	Example references
Biological and ecological	Shelter cats	Fewer cats into shelters	(53, 62, 124–126)
	Shelter cats	Reduced euthanasia of shelter cats/increased live release rate	(53, 62, 124–127)
	Free-roaming unowned cats	Improved welfare (e.g., feeding, prophylactic health care, morbidity, mortality)	(11, 84, 128)
	Free-roaming unowned cats	Behaviour changes (e.g., activity, urine spraying, aggression)	(35, 129)
	Free-roaming unowned cats	Reduction in numbers	(7, 11, 38, 84, 87, 92, 112)
	Free-roaming unowned cats	Stability	(35)
Social	Humans	Fewer complaints	(126, 127)
	Humans	Improved attitudes and behaviours	(36)
	Free-roaming unowned cats	Reduced noise disturbance or unwanted behaviours (e.g., mating calls, fighting, urine-spraying)	(130)
Methodological	Free-roaming unowned cats	No increase in mortality from procedure/safety or process	(44)
	Cost	Reduced cost	(10)

Practitioners often set the reduction of free-roaming unowned cat populations as a common goal and mark failure if these populations are not reduced (76, 81), which implicitly suggests this a realistic measure of success. However, only a limited number of studies successfully report population declines as an outcome (87–89). It can take time for population numbers to meaningfully reduce via natural-cause mortality and short-term success can be misleading as populations return due to compensatory processes. Additionally, long-term management is required due to people moving and changing residence within communities and cat ownership statuses changing, meaning unneutered owned cats will enter communities consistently (30). This may result in frustration among stakeholders if unrealistic expectations are held prior to programmes commencing. Indeed, where there are owned cats or neighbouring free-living, unowned cats, population management should be considered a permanent system of services that need to be sustained and adapted over time, rather than a short-term project.

Given cat populations are part of society, the ideal outcome for long-term impact may be one where management practices such as neutering become the social norm among community members, with owners and local residents developing a sense of community ownership over the problem, opposed to reactive delivery of TNR. Widespread engagement results in proactive reproduction control, in addition to other positive husbandry practices such as preventative or reactive healthcare, and resource provision where appropriate. In this regard, drivers of positive outcomes may move from being cat-centric to people-focussed goals.

## 8 Data gaps and considerations

There is a need to understand local cat populations and the communities they live in, as national or global solutions are not appropriate. Understanding the welfare, demographics and geographic distributions of cats within communities is crucial to inform operational priorities, set realistic goals, design appropriate interventions and reduce issues and geographic disparities associated with the management of cat populations. Here we describe some of the greatest gaps in our knowledge.

To understand and track numbers of unowned cats in the UK there is a need to work with stakeholders involved in cat population management to create and establish analytical and operational approaches that can differentiate between different subcategories of cats. This requires standardizing the way cat populations are termed and understood, to facilitate better data processing and use. In doing so, studies can investigate potential causes of overpopulation problems to establish and monitor links with predictors of abundance of unowned cats, including geographic, socioeconomic, environmental and cultural drivers.

Currently, very little is known about many aspects of unowned cat demographics globally, including in the UK. Improving our understanding of free-living, unowned cats in a UK context, with a focus on survival, reproduction, movement, density-dependent processes and carrying capacity is crucial data needed to build reliable population models and for integration into the development of management strategies. There is also a need to engage and work closely with homing organisations to improve our understanding on the demographics of cats in shelters, the impact of capacity for care and associated policies and processes. In doing so, practical evidence-based tools can be used to guide resource allocation in socially connected habitats as has been created in closed systems (108).

Moreover, there are limited UK studies on stakeholder views towards management options and expectations around outcomes based on realistic measures. Work is also needed to understand the balance between, and cat carer views towards, management options which target the individual versus the population. Management options for population benefit may mean difficult decisions at an individual level, and it is unclear the extent to which this is a barrier to effective population management in the UK.

## 9 Discussion

Management of unowned cats requires a multifaceted approach. Our aim is to synthesise the evidence base for management approaches of cat populations, including their ecology and the communities in which they reside. Undoubtedly, community and cultural differences will result in variation in how people respond to and engage with population management, making generalisations difficult. Through better understanding of the community links with overpopulation, targeted interventions are likely to be more successful.

The positive role shelters play in unowned cat management can be enhanced by cautious, optimal shelter intake, rather than open admission policies. While a full appraisal of how to optimise shelter management is beyond the scope of this review, in terms of intake, only cats that may benefit from being rehomed should be admitted. Shelters should not admit unsocialised or unadoptable cats as this may compromise individual welfare, as well as result in fewer cats being helped overall.

There is no standardised approach to evaluate cat population management, and measures of success must be realistic if managers are to avoid chronic failure. It must be acknowledged that although cats live in different circumstances, their subcategories are interconnected and subject to ecological processes that determine their density and carrying capacity. To prevent unrealistic expectations, management programmes must consider both ecological processes and societal norms of acceptance. Where owned cats are present, population management must be at scale and for the long-term. Given the ubiquitous and enduring presence of owned cats and neighbouring free-living, unowned cats, the ideal outcome is one where management practices such as neutering become the social norm among community members. Owners and residents engaging in community ownership over the problem is therefore more likely to bring about sustainable change compared to one-off interventions. While there is no one-size-fits-all solution to the management of unowned cats, long-term positive impact requires practitioners to consider the local community, environment and demographics of all cats, and to weigh the costs and benefits of different interventions using clear sets of acceptable and achievable targets. People that actively plan and participate in unowned cat management must think socially, economically and ecologically, consider both causes and symptoms of problems, and avoid the overly simplistic focus on controlling cat abundance.

## Footnotes

^1^Government of Norway. Animal Welfare Act. https://www.regjeringen.no/en/dokumenter/animal-welfare-act/id571188/ (2009).

^2^Cats Protection. Cats and Their Stats (CATS) UK 2024. (2024). https://www.cats.org.uk/media/hzfjahh2/cats-report-uk-2024.pdf (Accessed November 8, 2024).

^3^PDSA. PDSA Animal wellbeing report (PAW) 2024. (2024). https://www.pdsa.org.uk/what-we-do/pdsa-animal-wellbeing-report/paw-report-2024 (Accessed November 8, 2024).

^4^Cats Protection. The Veterinary Capacity Project from Cats Protection. https://www.cats.org.uk/media/vwmhzph2/cats-protection-vet-capacity-survey-report-2023.pdf (2023).

^5^UK Parliament H of CL. Research Briefing Rising cost of living in the UK. https://commonslibrary.parliament.uk/research-briefings/cbp-9428/ (Accessed November 13, 2024).

^7^The Cat Group. The Kitten Checklist. https://www.cats.org.uk/media/3722/the-kitten-checklist.pdf.

^8^Competition & Markets Authority. Veterinary Services for Household Pets in the UK. (2024). https://assets.publishing. service.gov.uk/media/65eee4015b6524420bf21a93/Veterinary_Services_for_Household_Pets_in_the_UK_-_Consultation_on_proposed_market_investigation_reference.pdf (Accessed November 25, 2024).

^9^International Cat Care. Cat Friendly Homing. https://icatcare.org/unowned-cats/cat-friendly-homing/ (Accessed November 13, 2024).

## References

[1] Gerhold RW Jessup DA. Zoonotic diseases associated with free-roaming cats. Zoonoses Public Health. (2013) 60:189–95. doi: 10.1111/j.1863-2378.2012.01522.x, PMID: 22830565

[2] Dabritz HA Conrad PA. Cats and toxoplasma: implications for public health. Zoonoses Public Health. (2010) 57:34–52. doi: 10.1111/j.1863-2378.2009.01273.x, PMID: 19744306

[3] Gunther I Raz T Berke O Klement E. Nuisances and welfare of free-roaming cats in urban settings and their association with cat reproduction. Prev Vet Med. (2015) 119:203–10. doi: 10.1016/j.prevetmed.2015.02.012, PMID: 25770734

[4] Loss SR Boughton B Cady SM Londe DW McKinney C O’Connell TJ. Review and synthesis of the global literature on domestic cat impacts on wildlife. J Anim Ecol. (2022) 91:1361–72. doi: 10.1111/1365-2656.13745 35593055

[5] Stavisky J Brennan ML Downes M Dean R. Demographics and economic burden of un-owned cats and dogs in the UK: results of a 2010 census. BMC Vet Res. (2012) 8:163. doi: 10.1186/1746-6148-8-163, PMID: 22974242 PMC3514250

[6] Cotterell J Rand J Scotney R. Rethinking Urban cat Management—limitations and unintended consequences of traditional cat Management. Animals. (2025) 15:1005. doi: 10.3390/ani15071005, PMID: 40218398 PMC11987726

[7] Natoli E Maragliano L Cariola G Faini A Bonanni R Cafazzo S. Management of feral domestic cats in the urban environment of Rome (Italy). Prev Vet Med. (2006) 77:180–5. doi: 10.1016/j.prevetmed.2006.06.005, PMID: 17034887

[8] Flockhart DTT Coe JB. Multistate matrix population model to assess the contributions and impacts on population abundance of domestic cats in urban areas including owned cats, unowned cats, and cats in shelters. PLoS One. (2018) 13:e0192139. doi: 10.1371/journal.pone.0192139, PMID: 29489854 PMC5830044

[9] McDonald J Finka L Foreman-Worsley R Skillings E Hodgson D. Cat: empirical modelling of *Felis catus* population dynamics in the UK. PLoS One. (2023) 18:e0287841. doi: 10.1371/journal.pone.0287841, PMID: 37437091 PMC10337951

[10] Benka VA Boone JD Miller PS Briggs JR Anderson AM Slootmaker C. Guidance for management of free-roaming community cats: a bioeconomic analysis. J Feline Med Surg. (2022) 24:975–85. doi: 10.1177/1098612X211055685, PMID: 34842477 PMC9511502

[11] Boone JD Miller PS Briggs JR Benka VAW Lawler DF Slater M. A long-term lens: cumulative impacts of free-roaming cat management strategy and intensity on preventable cat mortalities. Front Vet Sci. (2019) 6:238. doi: 10.3389/fvets.2019.00238, PMID: 31403048 PMC6676151

[12] Ramírez Riveros D González-Lagos C. Community engagement and the effectiveness of free-roaming cat control techniques: a systematic review. Animals. (2024) 14:492. doi: 10.3390/ani14030492, PMID: 38338135 PMC10854515

[13] McDonald JL Skillings E. Human influences shape the first spatially explicit national estimate of urban unowned cat abundance. Sci Rep. (2021) 11:20216. doi: 10.1038/s41598-021-99298-6, PMID: 34711904 PMC8553937

[14] Cecchetti M Crowley SL McDonald RA. Drivers and facilitators of hunting behaviour in domestic cats and options for management. Mamm Rev. (2021) 51:307–22. doi: 10.1111/mam.12230

[15] Doherty TS Dickman CR Johnson CN Legge SM Ritchie EG Woinarski JCZ. Impacts and management of feral cats *Felis catus* in Australia. Mamm Rev. (2017) 47:83–97. doi: 10.1111/mam.12080

[16] Rowan AN Kartal T Hadidian J. Cat demographics & impact on wildlife in the USA, the UK, Australia and New Zealand: facts and values. J Appl Anim Ethics Res. (2019) 2:7–37. doi: 10.1163/25889567-12340013

[17] Palmer A. The small British cat debate: conservation non-issues and the (im) mobility of wildlife controversies. Conserv Soc. (2022) 20:211–21. doi: 10.4103/cs.cs_92_21

[18] Lepczyk CA Fantle-Lepczyk JE Dunham KD Bonnaud E Lindner J Doherty TS. A global synthesis and assessment of free-ranging domestic cat diet. Nat Commun. (2023) 14:7809. doi: 10.1038/s41467-023-42766-6, PMID: 38086838 PMC10716121

[19] Philippe-Lesaffre M Bradshaw CJA Castañeda I Llewelyn J Dickman CR Lepczyk CA. Differential predation patterns of free-ranging cats among continents. Ecography. (2025) 2025:e07169. doi: 10.1111/ecog.07169

[20] Crawford C Rand J Forge O Rohlf V Bennett P Scotney R. A purr-suasive case for sterilization: how sterilizing working cats supports dairy farmers’ wellbeing, improves animal welfare, and benefits the environment. Animals. (2025) 15:766. doi: 10.3390/ani15060766, PMID: 40150295 PMC11939496

[21] Scotney R Rand J Rohlf V Hayward A Bennett P. The impact of lethal, enforcement-centred cat management on human wellbeing: exploring lived experiences of cat careers affected by cat culling at the port of Newcastle. Animals. (2023) 13:271. doi: 10.3390/ani13020271, PMID: 36670811 PMC9854822

[22] Kilshaw K Montgomery RA Campbell RD Hetherington DA Johnson PJ Kitchener AC. Mapping the spatial configuration of hybridization risk for an endangered population of the European wildcat (*Felis silvestris silvestris*) in Scotland. Mamm Res. (2016) 61:1–11. doi: 10.1007/s13364-015-0253-x

[23] Sainsbury KA Shore RF Schofield H Croose E Campbell RD Mcdonald RA. Recent history, current status, conservation and management of native mammalian carnivore species in Great Britain. Mamm Rev. (2019) 49:171–88. doi: 10.1111/mam.12150

[24] Breitenmoser U Lanz T Breitenmoser-Würsten C Conservation of the wildcat (*Felis silvestris*) in Scotland: review of the conservation status and assessment of conservation activities IUCN SSC cat specialist group (2019)

[25] Meredith A Bacon A Allan B Kitchener A Senn H Brooks S. Domestic cat neutering to preserve the Scottish wildcat. Vet Rec. (2018) 183:27–8. doi: 10.1136/vr.k2905, PMID: 29976717

[26] McDonald JL Farnworth MJ Clements J. Integrating trap-neuter-return campaigns into a social framework: developing long-term positive behavior change toward unowned cats in urban areas. Front Vet Sci. (2018) 5:258. doi: 10.3389/fvets.2018.00258, PMID: 30406118 PMC6207997

[27] Luzardo OP Hansen A Martín-Cruz B Macías-Montes A Travieso-Aja M d M. Integrating conservation and community engagement in free-roaming cat management: a case study from a Natura 2000 protected area. Animals. (2025) 15:429. doi: 10.3390/ani15030429, PMID: 39943199 PMC11815770

[28] Harkins C Shaw R Gillies M Sloan H Macintyre K Scoular A. Overcoming barriers to engaging socio-economically disadvantaged populations in CHD primary prevention: a qualitative study. BMC Public Health. (2010) 10:391. doi: 10.1186/1471-2458-10-391, PMID: 20598130 PMC2906468

[29] García Pinillos R Appleby MC Manteca X Scott-Park F Smith C Velarde A. One welfare – a platform for improving human and animal welfare. Vet Rec. (2016) 179:412–3. doi: 10.1136/vr.i5470, PMID: 27770094

[30] Cotterell J Rand J Scotney R. Urban cat Management in Australia—evidence-based strategies for success. Animals. (2025) 15:1083. doi: 10.3390/ani15081083, PMID: 40281917 PMC12024120

[31] McDowall S Hazel SJ Chittleborough C Hamilton-Bruce A Stuckey R Howell TJ. The impact of the social determinants of human health on companion animal welfare. Animals. (2023) 13:1113. doi: 10.3390/ani13061113, PMID: 36978653 PMC10044303

[32] Razee H Van Der Ploeg HP Blignault I Smith BJ Bauman AE McLean M. Beliefs, barriers, social support, and environmental influences related to diabetes risk behaviours among women with a history of gestational diabetes. Health Promot J Austr. (2010) 21:130–7. doi: 10.1071/he10130, PMID: 20701563

[33] Kelly S Martin S Kuhn I Cowan A Brayne C Lafortune L. Barriers and facilitators to the uptake and maintenance of healthy behaviours by people at mid-life: a rapid systematic review. PLoS One. (2016) 11:e0145074. doi: 10.1371/journal.pone.0145074, PMID: 26815199 PMC4731386

[34] Kennedy BPA Cumming B Brown WY. Global strategies for population management of domestic cats (*Felis catus*): a systematic review to inform best practice management for remote indigenous communities in Australia. Animals. (2020) 10:663. doi: 10.3390/ani10040663, PMID: 32290432 PMC7222776

[35] Cafazzo S Bonanni R Natoli E. Neutering effects on social behaviour of urban unowned free-roaming domestic cats. Animals. (2019) 9:1105. doi: 10.3390/ani9121105, PMID: 31835397 PMC6940995

[36] McDonald JL Clements J. Engaging with socio-economically disadvantaged communities and their cats: human behaviour change for animal and human benefit. Animals. (2019) 9:175. doi: 10.3390/ani9040175, PMID: 30999663 PMC6523136

[37] Dolan ED Weiss E Slater MR. Welfare impacts of spay/neuter-focused outreach on companion animals in New York City public housing. J Appl Anim Welf Sci. (2017) 20:257–72. doi: 10.1080/10888705.2017.1305904, PMID: 28481141

[38] Swarbrick H Rand J. Application of a protocol based on trap-neuter-return (TNR) to manage unowned urban cats on an Australian university campus. Animals. (2018) 8:77. doi: 10.3390/ani8050077, PMID: 29772788 PMC5981288

[39] Kessler M Von bomhard D. Mammary tumors in cats-epidemiologic and histologic features in 2386 cases (1990-1995). Kleintierpraxis. (1997) 42:459.

[40] Overley B Shofer FS Goldschmidt MH Sherer D Sorenmo KU. Association between ovarihysterectomy and feline mammary carcinoma. J Vet Intern Med. (2005) 19:560–3. doi: 10.1111/j.1939-1676.2005.tb02727.x, PMID: 16095174

[41] O’Neill DG Church DB McGreevy PD Thomson PC Brodbelt DC. Longevity and mortality of cats attending primary care veterinary practices in England. J Feline Med Surg. (2015) 17:125–33. doi: 10.1177/1098612X14536176 24925771 PMC10816413

[42] Brodbelt DC Blissitt KJ Hammond RA Neath PJ Young LE Pfeiffer DU. The risk of death: the confidential enquiry into perioperative small animal fatalities. Vet Anaesth Analg. (2008) 35:365–73. doi: 10.1111/J.1467-2995.2008.00397.X, PMID: 18466167

[43] Rigdon-Brestle K Accornero VH Amtower M Slater MR. Retrospective review reveals few complications of ovarian pedicle tie in 15,927 cats undergoing ovariohysterectomy at a large HQHVSN clinic and training facility in the United States: 2017–2018. J Am Vet Med Assoc. (2022) 260:S28–35. doi: 10.2460/JAVMA.21.09.0405, PMID: 35333751

[44] Levy JK Bard KM Tucker SJ Diskant PD Dingman PA. Perioperative mortality in cats and dogs undergoing spay or castration at a high-volume clinic. Vet J. (2017) 224:11–5. doi: 10.1016/j.tvjl.2017.05.013, PMID: 28697869

[45] Vester BM Sutter SM Keel TL Graves TK Swanson KS. Ovariohysterectomy alters body composition and adipose and skeletal muscle gene expression in cats fed a high-protein or moderate-protein diet. Animal. (2009) 3:91287–98. doi: 10.1017/s1751731109004868 22444905

[46] Belsito KR Vester BM Keel T Graves TK Swanson KS. Impact of ovariohysterectomy and food intake on body composition, physical activity, and adipose gene expression in cats. J Anim Sci. (2009) 87:594–602. doi: 10.2527/JAS.2008-0887, PMID: 18997063

[47] Häring T Haase B Zini E Hartnack S Uebelhart D Gaudenz D. Overweight and impaired insulin sensitivity present in growing cats. J Anim Physiol Anim Nutr (Berl). (2013) 97:813–9. doi: 10.1111/J.1439-0396.2012.01322.X, PMID: 22812383

[48] Welsh CP Gruffydd-Jones TJ Roberts MA Murray JK. Poor owner knowledge of feline reproduction contributes to the high proportion of accidental litters born to UK pet cats. Vet Rec. (2014) 174:118. doi: 10.1136/vr.101909, PMID: 24343905

[49] Murray JK Roberts MA Whitmarsh A Gruffydd-Jones TJ. Survey of the characteristics of cats owned by households in the UK and factors affecting their neutered status. Vet Rec. (2009) 164:137–41. doi: 10.1136/vr.164.5.137, PMID: 19188344

[50] Aguilar GD Farnworth MJ. Stray cats in Auckland, New Zealand: discovering geographic information for exploratory spatial analysis. Appl Geogr. (2012) 34:230–8. doi: 10.1016/j.apgeog.2011.11.011

[51] Finkler H Hatna E Terkel J. The influence of neighbourhood socio-demographic factors on densities of free-roaming cat populations in an urban ecosystem in Israel. Wildlife Res. (2011) 38:235–43. doi: 10.1071/WR10215

[52] Chu K Anderson WM Rieser MY. Population characteristics and neuter status of cats living in households in the United States. J Am Vet Med Assoc. (2009) 234:1023–30. doi: 10.2460/javma.234.8.1023, PMID: 19366332

[53] Cotterell J Rand J Barnes TS Scotney R. Impacts of a local government funded free cat sterilization program for owned and semi-owned cats. (2024). doi: 10.20944/preprints202403.0588.v1 PMC11171210 38891662

[54] Wongsaengchan C McKeegan DEF. The views of the UK public towards routine neutering of dogs and cats. Animals. (2019) 9:138. doi: 10.3390/ani9040138, PMID: 30986979 PMC6523704

[55] Günzel-Apel AR. Early castration of dogs and cats from the point of view of animal welfare. Dtsch Tierarztl Wochenschr. (1998) 105:95–8. 9581375

[56] Palmer C Corr S Sandøe P. Inconvenient desires: should we routinely neuter companion animals? Anthrozoös. (2012) 25:s153–72. doi: 10.2752/175303712x13353430377255

[57] Downes MJ Devitt C Downes MT More SJ. Neutering of cats and dogs in Ireland; pet owner self-reported perceptions of enabling and disabling factors in the decision to neuter. Peer J. (2015) 3:e1196. doi: 10.7717/peerj.1196, PMID: 26312187 PMC4548501

[58] Paiva MT Maia LD Franco Morais MH Nicolino RR Soares DF Oliveira CS. Enhancing access to dog and cat neutering Services in a City with a large population of stray animals. Prev Vet Med. (2025) 239:106491 Available at SSRN 4824074. doi: 10.2139/ssrn.4824074 40054333

[59] LaVallee E Mueller MK McCobb E. A systematic review of the literature addressing veterinary care for underserved communities. J Appl Anim Welf Sci. (2017) 20:381–94. doi: 10.1080/10888705.2017.1337515, PMID: 28657796

[60] Murray JK Mosteller JR Loberg JM Andersson M Benka VAW. Methods of fertility control in cats: owner, breeder and veterinarian behavior and attitudes. J Feline Med Surg. (2015) 17:790–9. doi: 10.1177/1098612x15594994, PMID: 26323804 PMC11148985

[61] Frank JM Carlisle-Frank PL. Analysis of programs to reduce overpopulation of companion animals: do adoption and low-cost spay/neuter programs merely cause substitution of sources? Ecol Econ. (2007) 62:740–6. doi: 10.1016/j.ecolecon.2006.09.011

[62] Levy JK Isaza NM Scott KC. Effect of high-impact targeted trap-neuter-return and adoption of community cats on cat intake to a shelter. Vet J. (2014) 201:269–74. doi: 10.1016/j.tvjl.2014.05.001, PMID: 24980808

[63] Robbins HJ Casey RA Clements J Gruffydd-Jones T Murray JK. Assessing the impact of a regional UK feline neutering campaign. Vet Rec. (2018) 182:291. doi: 10.1136/vr.104499, PMID: 29507110

[64] Kinsman RH Gruffydd-Jones TJ Clements J Murray JK. Risk factors for redemption of feline neutering vouchers issued by welfare organisations. Vet Rec. (2017) 181:427. doi: 10.1136/vr.104379, PMID: 28847874

[65] Murray JK Skillings E Gruffydd-Jones TJ. Opinions of veterinarians about the age at which kittens should be neutered. Vet Rec. (2008) 163:381–5. doi: 10.1136/vr.163.13.381, PMID: 18820325

[66] McDonald J Clements J. Contrasting practices and opinions of UK-based veterinary surgeons around neutering cats at four months old. Vet Rec. (2020) 187:317. doi: 10.1136/vr.105887, PMID: 32764034 PMC7606499

[67] Casey RA Bradshaw JWS. The effects of additional socialisation for kittens in a rescue centre on their behaviour and suitability as a pet. Appl Anim Behav Sci. (2008) 114:196–205. doi: 10.1016/j.applanim.2008.01.003

[68] Hargrave C. Helping kittens to become confident cats—why they and their owners need the support of the veterinary team. Part 2: environmental effects and support. Vet Nurse. (2018) 9:356–63. doi: 10.12968/vetn.2018.9.7.356

[69] Foreman-Worsley R Finka LR Ward SJ Farnworth MJ. Indoors or outdoors? An international exploration of owner demographics and decision making associated with lifestyle of pet cats. Animals. (2021) 11:253. doi: 10.3390/ani11020253, PMID: 33498511 PMC7909512

[70] Clancy EA Moore AS Bertone ER. Evaluation of cat and owner characteristics and their relationships to outdoor access of owned cats. J Am Vet Med Assoc. (2003) 222:1541–5. doi: 10.2460/javma.2003.222.1541, PMID: 12784959

[71] Tan SML Stellato AC Niel L. Uncontrolled outdoor access for cats: an assessment of risks and benefits. Animals. (2020) 10:258. doi: 10.3390/ani10020258, PMID: 32041155 PMC7070728

[72] Finka LR Foreman-Worsley R. Are multi-cat homes more stressful? A critical review of the evidence associated with cat group size and wellbeing. J Feline Med Surg. (2022) 24:65–76. doi: 10.1177/1098612X211013741, PMID: 34037488 PMC8807997

[73] Casey RA Vandenbussche S Bradshaw JWS Roberts MA. Reasons for relinquishment and return of domestic cats (*felis silvestris catus*) to rescue shelters in the UK. Anthrozoös. (2009) 22:347–58. doi: 10.2752/089279309X12538695316185

[74] Siettou C. Evaluating the recently imposed English compulsory dog microchipping policy. Evidence from an English local authority. Prev Vet Med. (2019) 163:31–6. doi: 10.1016/j.prevetmed.2018.12.015, PMID: 30670183

[75] Calver MC Crawford HM Fleming PA. Response to wolf et al.: furthering debate over the suitability of trap-neuter-return for stray cat management. Animals. (2020) 10:362. doi: 10.3390/ani10020362, PMID: 32102227 PMC7070824

[76] Crawford HM Calver MC Fleming PA. A case of letting the cat out of the bag—why trap-neuter-return is not an ethical solution for stray cat (*Felis catus*) management. Animals. (2019) 9:171. doi: 10.3390/ani9040171, PMID: 30995809 PMC6523511

[77] Wolf PJ Rand J Swarbrick H Spehar DD Norris J. Reply to Crawford et al.: why trap-neuter-return (TNR) is an ethical solution for stray cat management. Animals. (2019) 9:689. doi: 10.3390/ani9090689, PMID: 31527537 PMC6769729

[78] Read JL Dickman CR Boardman WSJ Lepczyk CA. Reply to Wolf et al.: why trap-neuter-return (tnr) is not an ethical solution for stray cat management. Animals. (2020) 10:1–10. doi: 10.3390/ani10091525 PMC7552220 32872227

[79] Barrows PL. Professional, ethical, and legal dilemmas of trap-neuter-release. J Am Vet Med Assoc. (2004) 225:1365–9. doi: 10.2460/javma.2004.225.1365, PMID: 15552310

[80] Jessup DA. The welfare of feral cats and wildlife. J Am Vet Med Assoc. (2004) 225:1377–83. doi: 10.2460/javma.2004.225.1377, PMID: 15552312

[81] Longcore T Rich C Sullivan LM. Critical assessment of claims regarding management of feral cats by trap-neuter-return. Conserv Biol. (2009) 23:887–94. doi: 10.1111/j.1523-1739.2009.01174.x, PMID: 19245489

[82] Kreisler RE Cornell HN Levy JK. Decrease in population and increase in welfare of community cats in a twenty-three year trap-neuter-return program in Key Largo, FL: the ORCAT program. Front Vet Sci. (2019) 6:7. doi: 10.3389/fvets.2019.00007, PMID: 30775368 PMC6367225

[83] Miller PS Boone JD Briggs JR Lawler DF Levy JK Nutter FB. Simulating free-roaming cat population management options in open demographic environments. PLoS One. (2014) 9:1–17. doi: 10.1371/journal.pone.0113553 PMC4245120 25426960

[84] Gunther I Hawlena H Azriel L Gibor D Berke O Klement E. Reduction of free-roaming cat population requires high-intensity neutering in spatial contiguity to mitigate compensatory effects. Proc Natl Acad Sci. (2022) 119:e2119000119. doi: 10.1073/pnas.2119000119, PMID: 35377788 PMC9169806

[85] Seo A Ueda Y Tanida H. Population dynamics of community cats living in a tourist area of Onomichi City, Japan, before and after the trap-test-vaccinate-alter-return-monitor event. J Appl Anim Welf Sci. (2023) 26:153–67. doi: 10.1080/10888705.2021.1901226, PMID: 33856958

[86] Kilgour RJ Magle SB Slater M Christian A Weiss E DiTullio M. Estimating free-roaming cat populations and the effects of one year trap-neuter-return management effort in a highly urban area. Urban Ecosyst. (2017) 20:207–16. doi: 10.1007/s11252-016-0583-8

[87] Spehar DD Wolf PJ. The impact of targeted trap–neuter–return efforts in the San Francisco bay area. Animals. (2020) 10:1–12. doi: 10.3390/ani10112089 PMC7698188 33187180

[88] Junqueira ANN Galera PD. Evaluation of population management based on trap–neuter–return and trap–neuter–adoption practices in a free-roaming cat colony in the Federal District, Brazil. Animals. (2024) 14:2478. doi: 10.3390/ani14172478, PMID: 39272261 PMC11394398

[89] Spehar DD Wolf PJ. An examination of an iconic trap-neuter-return program: the Newburyport, Massachusetts case study. Animals. (2017) 7:81. doi: 10.3390/ani7110081, PMID: 29088106 PMC5704110

[90] Ransom JI Powers JG Thompson Hobbs N Baker DL. Ecological feedbacks can reduce population-level efficacy of wildlife fertility control. J Appl Ecol. (2014) 51:259–69. doi: 10.1111/1365-2664.12166, PMID: 25558083 PMC4278530

[91] Foley P Foley JE Levy JK Paik T. Analysis of the impact of trap-neuter-return programs on populations of feral cats. J Am Vet Med Assoc. (2005) 227:1775–81. doi: 10.2460/javma.2005.227.1775, PMID: 16342526

[92] Tan K Rand J Morton J. Trap-neuter-return activities in urban stray cat colonies in Australia. Animals. (2017) 7:46. doi: 10.3390/ani7060046, PMID: 28574465 PMC5483609

[93] Lazenby BT Mooney NJ Dickman CR. Effects of low-level culling of feral cats in open populations: a case study from the forests of southern Tasmania. Wildlife Res. (2015) 41:407–20. doi: 10.1071/WR14030

[94] Palmas P Gouyet R Oedin M Millon A Cassan J-J Kowi J. Rapid recolonisation of feral cats following intensive culling in a semi-isolated context. NeoBiota. (2020) 63:177–200. doi: 10.3897/neobiota.63.58005

[95] Bradshaw JWS. Sociality in cats: a comparative review. J Vet Behav Clin Appl Res. (2016) 11:113–24. doi: 10.1016/j.jveb.2015.09.004

[96] Prentice JC Fox NJ Hutchings MR White PCL Davidson RS Marion G. When to kill a cull: factors affecting the success of culling wildlife for disease control. J R Soc Interface. (2019) 16:20180901. doi: 10.1098/rsif.2018.0901, PMID: 30836896 PMC6451411

[97] Miguel E Grosbois V Caron A Pople D Roche B Donnelly CA. A systemic approach to assess the potential and risks of wildlife culling for infectious disease control. Commun Biol. (2020) 3:353. doi: 10.1038/s42003-020-1032-z, PMID: 32636525 PMC7340795

[98] Finka LR. Conspecific and human sociality in the domestic cat: consideration of proximate mechanisms, human selection and implications for cat welfare. Animals. (2022) 12:298. doi: 10.3390/ani12030298, PMID: 35158622 PMC8833732

[99] Kessler MR Turner DC. Socialization and stress in cats (*Felis Silves tris catvs*) housed singly and in groups in animal shelters. Anim Welf. (1999) 8:15–26. doi: 10.1017/S0962728600021163

[100] Karsten CL Wagner DC Kass PH Hurley KF. An observational study of the relationship between capacity for care as an animal shelter management model and cat health, adoption and death in three animal shelters. Vet J. (2017) 227:15–22. doi: 10.1016/j.tvjl.2017.08.003, PMID: 29031325

[101] Janke N Berke O Flockhart T Bateman S Coe JB. Risk factors affecting length of stay of cats in an animal shelter: a case study at the Guelph humane society, 2011–2016. Prev Vet Med. (2017) 148:44–8. doi: 10.1016/j.prevetmed.2017.10.007, PMID: 29157373

[102] Jacobs J Reese LA. Compassion fatigue among animal shelter volunteers: examining personal and organizational risk factors. Anthrozoös. (2021) 34:803–21. doi: 10.1080/08927936.2021.1926719

[103] Riley S. The changing legal status of cats in Australia: from friend of the settlers, to enemy of the rabbit, and now a threat to biodiversity and biosecurity risk. Front Vet Sci. (2019) 5:342. doi: 10.3389/fvets.2018.00342, PMID: 30834250 PMC6387928

[104] Dabritz HA Atwill ER Gardner IA Miller MA Conrad PA. Outdoor fecal deposition by free-roaming cats and attitudes of cat owners and nonowners toward stray pets, wildlife, and water pollution. J Am Vet Med Assoc. (2006) 229:74–81. doi: 10.2460/javma.229.1.74, PMID: 16817717

[105] Hurley KF Levy JK. Rethinking the animal shelter’s role in free-roaming cat Management. Front Vet Sci. (2022) 9:847081. doi: 10.3389/fvets.2022.847081, PMID: 35372561 PMC8964341

[106] Clark CCA Gruffydd-Jones T Murray JK. Number of cats and dogs in UK welfare organisations. Vet Rec. (2012) 170:493. doi: 10.1136/vr.100524, PMID: 22589036

[107] Murray JK Skillings E Gruffydd-Jones TJ. A study of risk factors for cat mortality in adoption centres of a UK cat charity. J Feline Med Surg. (2008) 10:338–45. doi: 10.1016/j.jfms.2008.01.005, PMID: 18375164 PMC7129399

[108] Cecchetti M Nelli L. Planning and optimizing neutering programs for free-roaming cat populations: an interactive tool for cost-effective management in closed systems. J Appl Ecol. (2025) 62:1421–36. doi: 10.1111/1365-2664.70052

[109] Finkler H Gunther I Terkel J Finkler H Terkel J. Behavioral differences between urban feeding groups of neutered and sexually intact free-roaming cats following a trap-neuter-return procedure. J Am Vet Med Assoc. (2011) 238:1134–40. doi: 10.2460/javma.238.9.1134 21529235

[110] Kass PH Johnson KL Weng HY. Evaluation of animal control measures on pet demographics in Santa Clara County, California, 1993-2006. PeerJ. (2013) 1:e18. doi: 10.7717/peerj.18, PMID: 23638352 PMC3628371

[111] Nutter FB Levine JF Stoskopf MK. Reproductive capacity of free-roaming domestic cats and kitten survival rate. J Am Vet Med Assoc. (2004) 225:1399–402. doi: 10.2460/javma.2004.225.1399, PMID: 15552315

[112] Schmidt PM Lopez RR Collier BA. Survival, fecundity, and movements of free-roaming cats. J Wildl Manag. (2007) 71:915–9. doi: 10.2193/2006-066

[113] Devillard S Say L Pontier D. Dispersal pattern of domestic cats (*Felis catus*) in a promiscuous urban population: do females disperse or die? J Anim Ecol. (2003) 72:203–11. doi: 10.1046/j.1365-2656.2003.00692.x

[114] Ng TT Fascetti AJ Larsen JA. Reproduction of domestic cats in laboratories, catteries, and feral colonies: a review. Top Companion Anim Med. (2023) 55:100780. doi: 10.1016/j.tcam.2023.100780, PMID: 37225041

[115] Zito S Walker J Carolyn Gates M Dale A. A preliminary description of companion cat, managed stray cat, and unmanaged stray cat welfare in Auckland, New Zealand using a 5-component assessment scale. Front Vet Sci. (2019) 6:40. doi: 10.3389/fvets.2019.00040, PMID: 30854376 PMC6396406

[116] Jennett AL Jennett NM Hopping J Yates D. Evidence for seasonal reproduction in UK domestic cats. J Feline Med Surg. (2016) 18:804–8. doi: 10.1177/1098612X15595665, PMID: 26293245 PMC11112208

[117] Asa C Moresco A. Fertility control in wildlife: Review of current status, including novel and future technologies. Cham: Springer. (2019). 507–543 p. 10.1007/978-3-030-23633-5_17 31471808

[118] McDonald JL Hodgson D. Counting cats: the integration of expert and citizen science data for unbiased inference of population abundance. Ecol Evol. (2021) 11:4325–38. doi: 10.1002/ece3.7330, PMID: 33976813 PMC8093703

[119] Helback O Liebezeit J. Density of free-roaming cats related to feeding stations on Hayden Island, Oregon; (2021). doi: 10.15760/honors.1005

[120] Tennent J Downs CT. Abundance and home ranges of feral cats in an urban conservancy where there is supplemental feeding: a case study from South Africa. Afr Zool. (2008) 43:218–29. doi: 10.3377/1562-7020-43.2.218

[121] Hernandez SM Loyd KAT Newton AN Carswell BL Abernathy KJ. Activity patterns and interspecific interactions of free-roaming, domestic cats in managed trap-neuter-return colonies. Appl Anim Behav Sci. (2018) 202:63–8. doi: 10.1016/j.applanim.2018.01.014

[122] Boone JD. Better trap–neuter–return for free-roaming cats. J Feline Med Surg. (2015) 17:800–7. doi: 10.1177/1098612X15594995, PMID: 26323805 PMC11148983

[123] Marino F McDonald RA Crowley SL Hodgson DJ. Rethinking the evaluation of animal translocations. Biol Conserv. (2024) 292:110523. doi: 10.1016/j.biocon.2024.110523

[124] Hamilton F. Implementing nonlethal solutions for free-roaming cat management in a county in the southeastern United States. Front Vet Sci. (2019) 6:259. doi: 10.3389/fvets.2019.00259, PMID: 31508428 PMC6714295

[125] Johnson KL Cicirelli J. Study of the effect on shelter cat intakes and euthanasia from a shelter neuter return project of 10,080 cats from march 2010 to June 2014. PeerJ. (2014) 2:e646. doi: 10.7717/peerj.646, PMID: 25374785 PMC4217190

[126] Rand J Saraswathy AM Verrinder J Paterson MBA. Outcomes of a community cat program based on sterilization of owned, semi-owned and unowned cats in a small rural town. Animals. (2024) 14:3058. doi: 10.3390/ani14213058, PMID: 39518781 PMC11545350

[127] Hughes KL Slater MR Haller L. The effects of implementing a feral cat spay/neuter program in a Florida county animal control service. J Appl Anim Welf Sci. (2002) 5:285–98. doi: 10.1207/S15327604JAWS0504_03, PMID: 16221079

[128] Gunther I Raz T Zor YE Bachowski Y Klement E. Feeders of free-roaming cats: personal characteristics, feeding practices, and data on cat health and welfare in an urban setting of Israel. Front Vet Sci. (2016) 3:21. doi: 10.3389/fvets.2016.00021, PMID: 27014704 PMC4779851

[129] Finkler H Hatna E Terkel J. The impact of anthropogenic factors on the behavior, reproduction, management and welfare of urban, free-roaming cat populations. Anthrozoös. (2011) 24:31–49. doi: 10.2752/175303711X12923300467320

[130] Scarlett J Johnston N. Impact of a subsidized spay neuter clinic on impoundments and euthanasia in a community shelter and on service and complaint calls to animal control. J Appl Anim Welf Sci. (2012) 15:53–69. doi: 10.1080/10888705.2012.624902, PMID: 22233215

